# Could HE4 level measurements during first-line chemotherapy predict response to treatment among ovarian cancer patients?

**DOI:** 10.1371/journal.pone.0194270

**Published:** 2018-03-27

**Authors:** Anita Chudecka-Głaz, Aneta Cymbaluk-Płoska, Małgorzata Wężowska, Janusz Menkiszak

**Affiliations:** Department of Gynecological Surgery and Gynecological Oncology of Adults and Adolescents, Pomeranian Medical University, Szczecin, Poland; University of South Alabama Mitchell Cancer Institute, UNITED STATES

## Abstract

**Background:**

This study assessed the prognostic value of HE4 marker measurements at various stages of first-line chemotherapy for ovarian cancer.

**Methods:**

The study consisted of 90 ovarian cancer patients, including 48 women undergoing primary surgical treatment and 42 patients qualified for neoadjuvant chemotherapy. Each patient underwent HE4 and CA 125 level measurements at the time of diagnosis and subsequently as follows: after surgical treatment, after the third course of adjuvant chemotherapy, before interval cytoreductive surgery and after chemotherapy. The HE4 value was assessed based on the PSF, OS, DFS, surgical outcome, two-year survival and platinum sensitivity.

**Results:**

Preoperative HE4 levels were a predictor of platinum sensitivity (AUC– 0.644; p = 0.035) and DFS (AUC = 0.637; p = 0.0492). A univariate logistic regression analysis showed that serum HE4 significantly correlated with PFS (baseline results over median HR = 2.96, p = 0.0009; baseline over 75 percentile HR = 2.44, p = 0.0062; normalization after treatment HR = 0.46, p = 0.0125; 50% reduction before IDS HR = 0.64, p = 0.0017). In the multivariate analysis, normalization after treatment and 50% reduction before IDS significantly influenced the PFS (HR = 0.29, p = 0.00008; HR = 0.23, p = 0.0024). The HE4 levels also correlated with the OS as follows: values below the median (HR = 1.88, p = 0.0087), normalization after chemotherapy (HR = 0.08, p = 0.0003), and 50% reduction before IDS (HR = 0.39, p = 0.0496).

**Conclusions:**

The significant effect of the normalization of the HE4 marker after therapy and 50% reduction of HE4 levels before interval cytoreductive surgery on PFS and OS confirmed that HE4 might be an independent prognostic factor of treatment response. HE4 measurements performed during first-line treatment of ovarian cancer may have prognostic significance.

## Introduction

Despite the use of most modern treatments, ovarian cancer is characterized with the worst survival parameters among all gynecological malignancies. As a standard of adjuvant treatment in Poland according to the recommendations of the Polish Society of Gynecologic Oncology, platinum derivatives in combination with paclitaxel are used as a first-line treatment [1]. This treatment is concordant with the protocols of ovarian cancer treatment in other European countries [2]. The effects of the treatment are somewhat better in the recent years but are still unsatisfactory due to highly frequent recurrences, despite achieving primary complete remission. Only approximately 10%-15% of patients with advanced ovarian cancer reach extended, several-year-long disease-free periods [3]. A greater proportion of women achieving long periods of complete remission increased by 38% in breast cancer and by only 17% in ovarian cancer [4]. It seems that a significant number of these failures results from resistance to primary treatment which, in the case of ovarian cancer, is related to platinum resistance. Approximately 20% of patients beginning treatment are resistant to platinum therapy and will either not respond to the treatment at all, have disease progression during chemotherapy, or will present with early relapse within 6 months from the end of chemotherapy. The second important factor affecting the survival of patients is optimal, and in today's era, complete cytoreduction at the initial operation. Therefore, predicting a surgical outcome based on biomarker behavior can provide very valuable information.

For that reason, there is a need for finding suitable biomarkers that might predict platinum sensitivity, surgical outcome and finally survival parameters.

At present, more than 2,000 works concerning CA 125 have been published, and only 370 related to HE4. The assessment of the prognostic significance of CA 125 has been described in more than 1000 scientific papers, whereas in the case of HE4 such works are only about 100. HE4 is still a marker whose significance of diagnostic and prognostic value must be thoroughly investigated.

The goal of this publication was to determine whether the HE4 protein might serve as a biomarker for the response to treatment among patients with ovarian cancer subjected to first-line chemotherapy based on platinum analogues.

## Materials and methods

### Study population

Ethics permission for the study was approved by the Bioethics Committee of Pomeranian Medical University (protocol number KB-0012/58/11). All patients gave written consent. This report describes a prospective study. Initially, the study included patients with suspected ovarian cancer (118 women) referred for laparoscopy or laparotomy depending on disease advancement, general health status, the local status determined based on gynecological examination and computed tomography. Following confirmation of the diagnosis of malignant epithelial ovarian cancer by histopathological examination, the patients were qualified for further stages of the study. Five women with germinal and gonadal ovarian tumors diagnosed in histopathological examination were excluded. The study continued with 113 patients with ovarian cancer, who had commenced chemotherapy. However, another 23 patients were rejected due to refusal of chemotherapy, change of treatment facility, death due to non-oncological reasons or lack of follow-up after the end of treatment. Finally, 90 women with all of the data necessary for proper analysis were included in the study. Table 1 demonstrates the patients’ characteristics. All of the patients signed an informed consent to participate in the study.

**Table 1:** Clinicopathological characteristics of the patients with ovarian cancer (n = 90).

Characteristics	All groups n = 90	Primary debulking surgery (PDS) n = 48	HE4 (PDS) [pmol/l] Median (range)	Neoadjuvant chemotherapy (NACT) n = 42	HE4 (NACT) [pmol/l] Median (range)
Age, median (range)					
all	60,4 (31–87)	58.6 (31–81)	307.1 (35.8–3608)	59,8 (33–87)	-
premenopausal	43,6 (31–51)	40,8 (31–50)	98.3 (35.8–233)	46 (38–51)	577.5 (111–116.3)
postmenopausal	60,6 (48–87)	60,6 (48–81)	324.4 (41.1–1500)	65,2 (52–87)	820 (53.1–3608)
FIGO stage, n (%)					
I and II	17 (18.9)	17 (35.4)	139.2 (41.1–345)	-	-
III and IV	73 (81.1)	31 (64.6)	594.8 (35.8–1500)	42 (100)	827.9 (53.1–3608)
Histology, n (%)					
Serous	80 (88.9)	38 (79.2)	354.8 (35.8–850)	42 (100)	827.9 (53.1–3608)
Mucinous	7 (7.8)	7 (14.6)	109.3 (48.4–246.1)	-	-
Endometrioid	3 (3.3)	3 (6.2)	120.7 (65.8–121)	-	-
Tumor grade, n (%)					
1	17 (18.9)	12 (25)	101.8 (35.8–1441)	5 (11.9)	384.5 (111–658.4)
2	24 (26.7)	13 (27.1)	233 (65.8–1500)	11 (26.2)	427.5 (53.9–116.3)
3	49 (54.4)	23 (47.9)	427.5 (41–1500)	26 (61.9)	897.4 (53.3–3608)
Surgery, n (%)					
Optimal	65 (72.2)	37 (77.1)	186.3 (35.8–850)	28 (70)	817.6 (53.1–3608)
Suboptimal	25 (27.8)	11 (22.9)	708.7 (65.9–1500)	14 (30)	785.5 (53.9–2556)

Among the 90 women, 48 subjects received adjuvant therapy after primary cytoreductive surgery (PDS), and after subsequent laparoscopy or exploratory laparotomy, 42 patients were referred for neoadjuvant treatment (NACT). The number of treatment courses in adjuvant therapy amounted to six, while as a part of neoadjuvant therapy patients received 3–4 courses before surgery (IDS—interval debulking surgery) and 5–6 courses after surgical treatment.

### Analysis of serum HE4 and CA 125

Blood for HE4 and CA 125 was drawn on the first day the patients reported to the hospital and thus before the final diagnosis. The so-called baseline level of markers was assessed, and they were drawn before each subsequent chemotherapy course (for the final analysis, we used the level after the primary cytoreductive surgery and after the third course of chemotherapy), before the IDS surgery and after the end of treatment. All of the assessments were made immediately (within 2–3 hours) without the need for freezing and storing the material.

The HE4 serum levels of the marker were determined using the Roche Elecsys^®^ assay on a Cobas e601 apparatus. This assay is a one-step sandwich electro-chemiluminescence immunoassay (ECLIA) for the quantitative determination of human epididymal protein 4. The detection range for HE4 was 15.0–1500 pmol/L; in case of values exceeding 1500 pmol/L, the samples were diluted in a 1:20 ratio using Elecsys Diluent. The CA 125 serum levels of the marker were determined using the ARCHITECT CA 125 II assay on an ARCHITECT 2200SR System. This assay is a two-step immunoassay to determine the presence of CA 125 antigen using Chemiluminescent Microparticle Immunoassay (CMIA) technology. The CA 125 and HE4 assays were carried out according to the manufacturers' instructions, with the appropriate controls testing within the normal ranges.

### Evaluation of treatment response

The following parameters were used to assess the effectiveness of the treatment: PFS (progression-free survival), DFS (disease-free survival), two-year survival, OS (overall survival), and platinum-resistance assessment according to the following definitions:

- platinum sensitive patients, if the disease-free time period was longer than 6 months after the end of first-line chemotherapy.
- platinum resistant patients, if the disease-free time period was shorter than 6 months after the end of first-line chemotherapy.
- platinum refractory patients, if the disease progression occurred during first–line chemotherapy
- PFS—time from the initial diagnosis to recurrence of the disease or death, whichever came first
- DFS—time from the end of chemotherapy to relapse
- OS—time from the initial diagnosis to death, last contact or loss of follow-up.

Platinum resistant and platinum refractory patients were defined as non-responders to first-line chemotherapy. The elevation of markers during chemotherapy was an indication for computed tomography and its positive result—for diagnosis of disease progression. After the end of chemotherapy, the patients were followed up in the outpatient clinic. When the first clinical signs that might be associated with disease relapse appeared, the CA 125 and HE4 markers were assessed and, in the case of finding their increased levels relative to the previous values measured after the completion of therapy, computed tomography was performed. We used the RECIST 1.1 criteria [5] in the computed tomography assessment, while the analysis of CA 125 behavior was conducted on the basis of the GCIG guidelines as described by Rustin et al. [6]. A residual size of <1 cm was considered an optima cytoreduction, while a suboptimal cytoreduction was diagnosed with a residual mass over 1 cm.

### Statistical analysis

All of the statistical analyses were conducted using Statistica 10 software. Marker levels in particular groups and subgroups are demonstrated as the medians and ranges. Due to a lack of normal distribution, comparisons between the groups were performed with non-parametric U Mann-Whitney test. To assess the influence of HE4 and CA 125 markers on the overall survival or disease recurrence and time to progression, we used a Kaplan-Meier survival curve and a log rank test. For that purpose, we established cut-off points for the HE4 and CA 125, including the median baseline value of the markers, the 75 percentile of the baseline values, the 50% reduction of the markers after surgical treatment in the group after primary cytoreduction, normalization of the markers after the third course of chemotherapy in the patients after PDS, normalization of the markers after chemotherapy and in the group receiving neoadjuvant treatment a 50% reduction of markers before IDS as well as values over and below the median of the measurements performed before IDS. Uni- and multivariate analyses using the Cox regression model were performed. Parameters included in the multivariate Cox analysis included age, FIGO stage, grade, residual disease status and variants of serum HE4 and CA 125 concentrations. Age was assessed as a continuous variable, FIGO staging was divided into I/II vs. III/IV, grade G1+G2 vs. G3, residual disease into optimal vs. suboptimal surgery. Values of markers were included in the multivariate analysis according to the abovementioned criteria (cut-off points). The area under the curve (AUC) was calculated with a receiver operating characteristics (ROC) analysis and a logistic regression model was used to evaluated the markers’ ability to predict patient outcome, including the response to platinum therapy (responders vs. non-responders), relapse or obtaining/not obtaining two-year survival. We used logistic regression model as the maximum likelihood method. The results were considered to be significant for p<0.05.

## Results

### Patients, biomarkers baseline characteristics and correlation with classical prognostic factors

Table 1 presents the characteristics of the study group, as well as the HE4 marker values in the analyzed group and subgroups.

The median duration of the PFS was 27.8 (9–53) months in the entire study group, 31 (9–56) months in the group subjected to primary surgical treatment and 23.8 (4–40) months in the group receiving neoadjuvant therapy. The overall survival time equaled, respectively, 32.8 (3–56) months in the entire study population, 34.1 (6–56) in the group after PDS and 30.6 (3–54) months in the NACT group. Four patients progressed from the beginning of the treatment. The two-year survival was obtained in 72% (85% in the PDS and 67.1% in the NACT group) of the women, 70% were platinum-sensitive (79% in the PDS group and 59.5% in the NACT group), while DFS could not be obtained in 24.4% of the patients (14.5% in the PDS group and 35.7% in the NACT group).

Table 2 presents the values and comparisons of the HE4 and CA 125 marker values, depending on the typical prognostic factors in the entire study population.

**Table 2:** Comparison of the median levels of serum HE4 and CA 125 according to typical prognostic factors, PFS, OS and platinum-sensitivity in all the examined patients (Mann-Whitney U test).

Prognostic factor	HE4 [pmol/l]	CA125 [U/ml]
	Median [range]	Median [range]
Age		
Premenopausal, n = 12	172 [35.8–1116.3]	114.2 [33–4638]
Postmenopausal, n = 78	311 [41.1–3608]	323.5 [11.3–14199]
p-value	p = 0.1661	p = 0.9609
FIGO stage		
I and II, n = 17	120.7 [41.1–345]	74.5 [11.3–1441]
III and IV, n = 73	543 [35.8–3608]	535.1 [15.9–14199]
p-value	p = 0.000001	p = 0.000002
Tumor grade		
1 and 2, n = 41	226 [35.8–1500]	198 [11.3–1639]
3 n = 49	521 [41.1–2556]	521.8 [15.9–14199]
p-value	p = 0.0026	p = 0.0169
Histology		
Serous, n = 80	345 [35.9–3608]	371 [15.9–14199]
Mucinous, n = 7	109.8 [48.4–246.1]	110.2 [11.3–227.6]
p-value	p = 0.0019	p = 0.0003
Surgery		
Optimal, n = 65	226 [35.8–3608]	198.5 [11.3–14199]
Suboptimal, n = 25	543 [53.9–2556]	543 [20.4–10000]
p-value	p = 0.0047	p = 0.0004
DFS		
Yes, n = 68	242.5 [35.8–1500]	264.6 [11.3–14199]
No, n = 22	389.9 [53.9–3608]	536.5 [20.4–1000]
p-value	p = 0.0429	p = 0.0171
2-year survival		
Yes, n = 65	239 [35.8–1500]	227.6 [11.3–10000]
No, n = 25	385.2 [53.9–3608]	536 [20.4–14199]
p-value	p = 0.1289	p = 0.0269
Platinum sensitive, n = 63	239 [35.7–1500]	215 [11.3–14199]
Platinum resistant, n = 27	455 [53.9–3608]	535 [20.4–1000]
p-value	p = 0.0354	p = 0.018

Comparing the age, disease advancement, cellular diversity, histopathological type, surgical outcome, presence of clinical remission, two-year survival and platinum sensitivity, we noted statistically significant differences for both HE4 and CA 125 in almost all of the cases. Only in the assessment of two-year survival were there no differences in the median values with regard to HE4 between the patients who survived or did not survive two years from the diagnosis.

### HE4 and CA 125 in predicting surgical outcome, platinum resistance, surgical outcome, two-year survival and disease-free survival (DFS)

**Fig 1:**
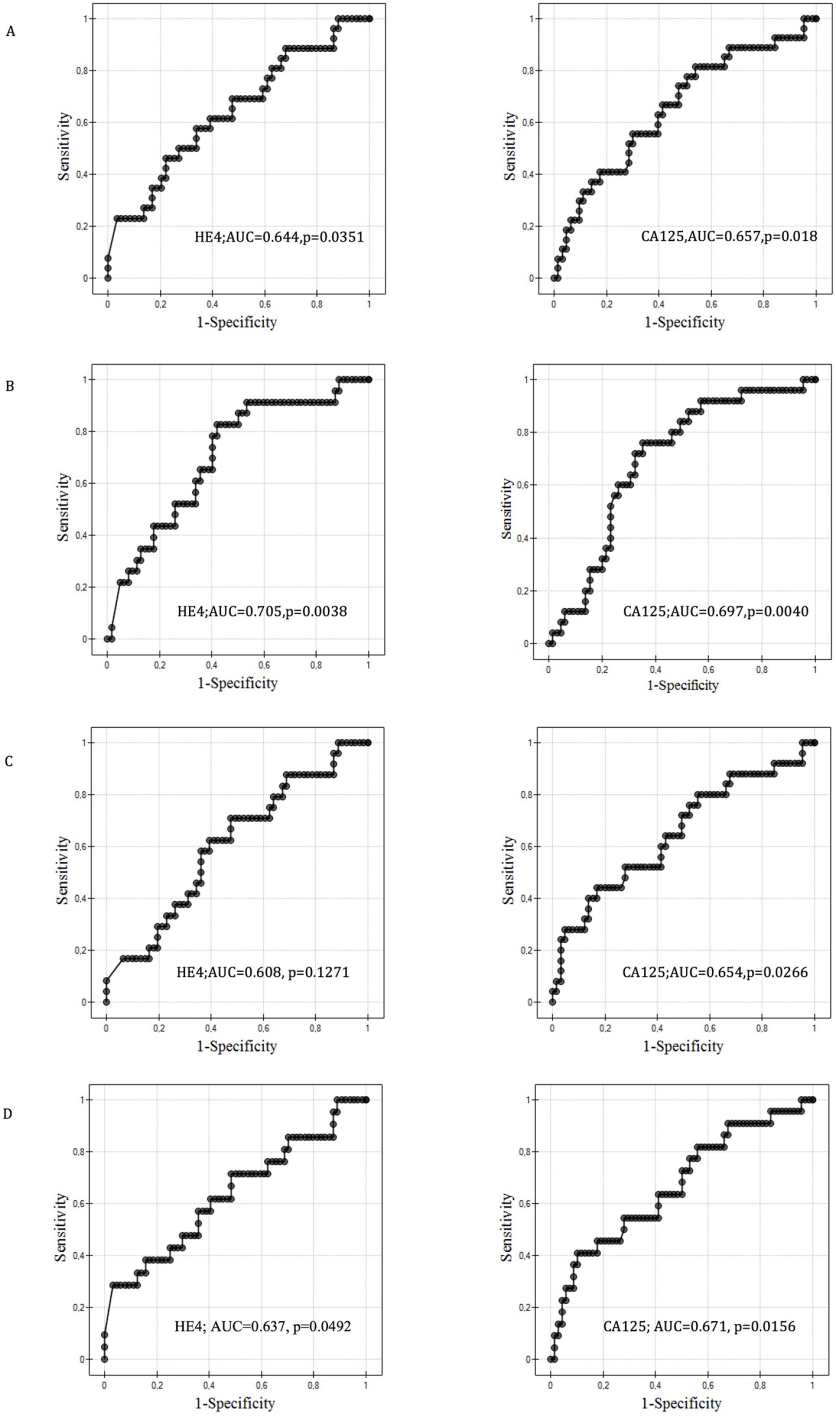
ROC of the plasma HE4 and CA125 for the prediction of maximal cytoreduction, platinum sensitivity, two-year survival and disease-free survival. (A) Platinum sensitive vs. platinum resistant. (B) Maximal cytoreduction vs. suboptimal debulking. (C) Reached two-year survival vs. did not reach two-year survival. (D) Reached disease-free survival vs. did not reach disease-free survival.

To determine the predictive capabilities of HE4 and CA 125 obtained prior to the start of the therapy, we used two statistical methods: calculated the ROC curves and assessed the value of the area under the curve (AUC), Fig 1 and traded on the logistic regression model.

Among the analyzed patients, 63 were considered platinum-sensitive (responders) and 27 were platinum—refractory and platinum-resistant (non-responders). The predictive abilities with regard to platinum sensitivity for HE and CA 125 were the following: HE4 AUC = 0.644 for all the patients, HE4 AUC = 0.627 for the patients after PDS and HE4 AUC = 0.53 for the patients treated with neoadjuvant chemotherapy. The respective values for CA 125 were 0.657, 0.547 and 0.619. From a group of 90 patients, 65 underwent optimal cytoreductive surgery, and 25 were suboptimal. An assessment of surgical outcome using the analyzed parameters in our material yielded the following results: HE4 AUC = 0.705 for all of the patients; HE4 AUC = 0.846 for the patients after primary surgical treatment and HE4 AUC = 0.452 for the patients receiving neoadjuvant chemotherapy. For the compared CA 125 markers, the AUC values were 0.697, 0.819 and 0.471. The assessment of the ability to predict the two-year survival among the patients with ovarian cancer using HE4 yielded the following results: HE4 AUC 0.608 for all the 90 studied subjects, HE4 AUC 0.479 for the patients after surgical treatment, HE4 AUC = 0.498 for the NACT group, and for CA 125, 0.654, 0.33 and 0.681, respectively. Initially, 68 women obtained disease-free survival (DFS), and 22 patients presented with no remission. The ability to predict DFS with HE4 and CA 125 markers assessed by ROC and AUC were as follows: HE4 AUC = 0.637 for all of the patients; HE4 AUC = 0.586 for the surgically treated patients and HE4 AUC = 0.52 for the NACT group. The area under the curves for CA 125 in the measured parameter amount were, respectively, 0.671, 0.537 and 0.617.

### HE4 and CA 125 impact on OS and PFS

The studied cohort of patients was subjected to dichotomization based on the HE4 and CA 125 levels measured in various stages of patient treatment:

- The median value of the initial marker levels (HE4 = 307 pmol/L; CA 125 = 683 mIU/L),
- 75th percentile of the initial marker levels (HE4 = 683 pmol/L; CA 125 = 682 mIU/L),
- 50% reduction (or lack thereof) in the marker levels after surgery in the group of patients who received primary surgical treatment (PDS),
- normalization of the marker levels after the 3^rd^ course of chemotherapy among the patients after primary surgical treatment,
- 50% reduction (or lack thereof) in the marker levels before interval debulking surgery in the group of patients after neoadjuvant treatment (NACT),
- normalization of the markers after the end of treatment—the last chemotherapy.

**Fig 2:**
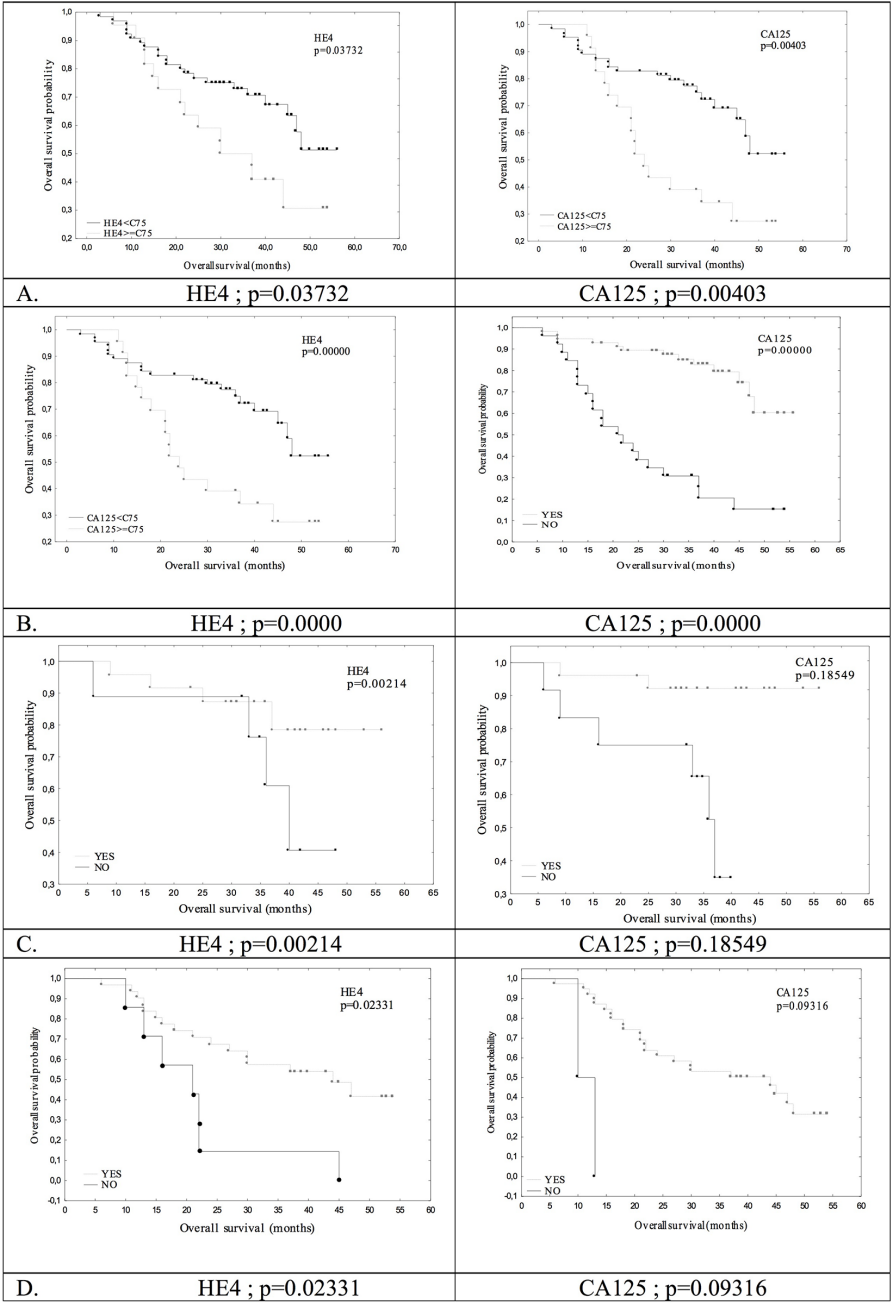
Overall survival stratified by HE4 and CA125 in the examined patients with ovarian cancer. (A) Stratified by preoperative serum marker levels (75 percentile) in the whole group. (B) Stratified by normalization after the last course of chemotherapy in the whole group. (C) Stratified by normalization of the serum marker levels after 3 courses of chemotherapy in patients after the primary debulking surgery. (D) Stratified by the 50% reduction of serum marker levels before the interval debulking surgery.

**Fig 3:**
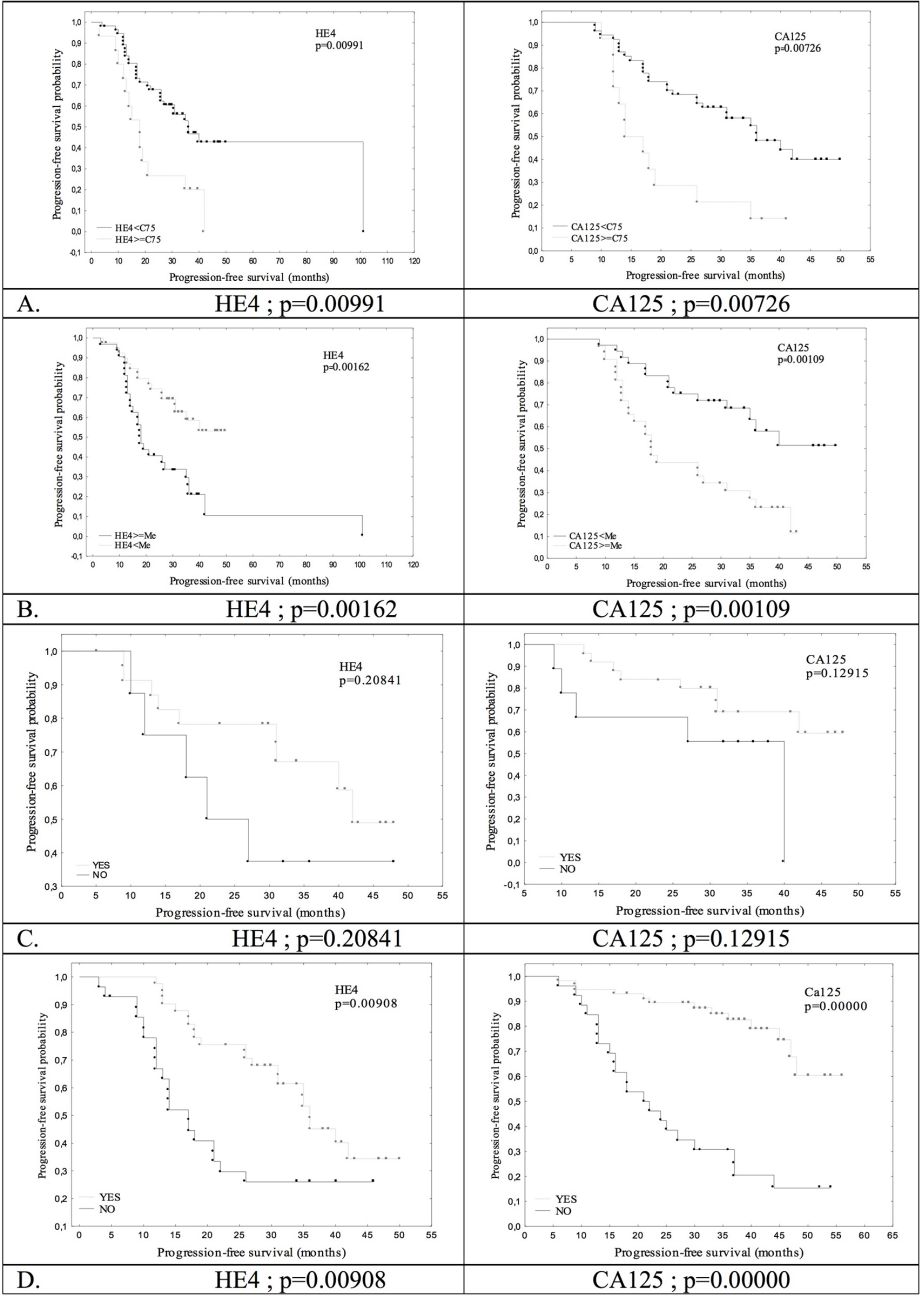
Progression-free survival stratified by HE4 and CA125 in the examined patients with ovarian cancer. (A) Stratified by the preoperative serum marker levels (75 percentile) in the whole group. (B) Stratified by the preoperative serum marker levels (median) in the whole group. (C) Stratified by the 50% reduction of serum marker levels after the surgical treatment in patients after the primary debulking surgery. (D) Stratified by normalization after the last course of chemotherapy in the whole group.

The survival analysis was conducted based on the Kaplan-Meier curves and log rank comparisons, and the most interesting results are presented on Figs 2 and 3.

The duration of PFS was significantly influenced by the values of both neoplastic markers before treatment (median and 75. percentile) and the normalization of the marker levels after the end of therapy. However, there was no impact of the 50% reduction in HE4 and CA 125 levels after the first surgical treatment, the normalization of the HE4 levels after the third course of chemotherapy (p = 0.5618) and the 50% reduction in CA 125 (p = 0.2856) and HE4 (p = 0.1215) levels before the interval cytoreductive surgery. The normalization of the CA 125 concentration after the 3^rd^ course of postoperative chemotherapy significantly correlated with the prolonged PFS (p = 0.0154). The impact of the analyzed HE4 marker on the overall patient survival (OS) was significant in almost all of the analyzed subgroups after dichotomization, except for the 50% reduction after surgery (p = 0.2084). The preoperative levels of CA 125 (median; p = 0.0425, 75. percentile), the normalization of the values after last chemotherapy and a 50% reduction after surgery (p = 0.0051) had an impact on the OS. The overall survival was not influenced by the normalization of the CA 125 marker after the third course or by the 50% reduction before IDS.

We conducted univariate and multivariate Cox regression analyses in order to assess the influence of widely recognized, standard prognostic factors, as well as the levels of the HE4 marker measured and analyzed during the treatment on PFS and OS (Table 3).

**Table 3:** Cox regression analyses of the progression-free survival and overall survival of classic prognostic factors and HE4.

	Univariate analysis (Cox regression model)					
	PFS			OS		
	HR	95% CI	p-value	HR	95% CI	p-value
Age	1.02	0.99–1.05	0.1538	1.05	1.01–1.08	0.0045
Stage I, II vs. III, IV	3.59	1.65–7.81	0.0013	4.64	1.41–15.3	0.0115
Grade 1,2 vs. 3	1.54	0.83–2.66	0.17	1.12	0.55–2.26	0.7607
Residual disease O-1 vs. >1	2.29	1.18–4.44	0.0141	1.45	0.69–3	0.3209
Histopathology serous vs. other	2.66	0.9507.49	0.0634	0.97	0.37–2.53	0.9515
HE4–median before diagnosis	2.96	1.56–5.62	0.0009	1.88	0.91–3.88	0.0087
HE4–75 percentile before diagnosis	2.44	1.26–4.74	0.0062	1.77	0.84–3.7	0.1287
HE4-normalization after chemotherapy	0.46	0.25–0.84	0.0125	0.08	0.02–0.25	0.0003
HE4- 50% reduction after surgery (PDS)	0.64	0.21–1.95	0.4361	0.21	0.04–1.07	0.0604
HE4-50% reduction before IDS (NACT)	0.25	0.11–0.59	0.0017	0.39	0.16–0.98	0.0469
HE4-normalisation after 3 course of cht. (PDS)	1.38	0.45–4.15	0.5698	0.99	0.22–4.47	0.9966
	Multivariate analysis (Cox regression model)					
	PFS			OS		
	HR	95% CI	p-value	HR	95% CI	p-value
Age	1.48	0.56–3.89	0.4287	1.04	1.0–1.07	0.0137
Stage I, II vs. III, IV	2.88	1.21–6.85	0.0164	5.24	1.47–18.61	0.0105
Grade 1,2 vs. 3	1.42	0.73–2.77	0.3033	0.96	0.46–1.99	0.9156
Residual disease O-1 vs. >1	3.9	1.28–12.21	0.0471	0.9	0.41–1.95	0.7912
Histopathology serous vs. other	1.77	0.6–5.26	0.2984	0.49	0.18–1.42	0.1925
HE4 –median before diagnosis	1.49	0.66–3.36	0.3367	1.97	0.81–4.83	0.1344
HE4–75 percentile before diagnosis	1.17	0.54–2.52	0.698	1.25	0.55–2.85	0.5846
HE4-normalization after chemotherapy	0.29	0.15–0.61	0.00008	0.08	0.02–0.27	0.00005
HE4- 50% reduction after surgery (PDS)	0.7	0.18–2.69	0.6068	0.44	0.06–2.83	0.389
HE4-50%reduction before IDS (NACT)	0.23	0.09–0.59	0.0024	0.59	0.19–1.78	0.3464
HE4-normalisation after 3 course of cht. (PDS)	2.53	0.69–9.28	0.1601	0.32	0.05–2.04	0.2297

We found in both the univariate and the multivariate analyses that among all of the selected prognostic factors, the degree of clinical advancement and residual disease exerted the strongest influence on PFS. However, age and the degree of advancement of malignant disease exerts the greatest influence on overall survival. Among the analyzed concentrations of the HE4 marker, the univariate analysis demonstrated a significant influence of HE4 on the selected survival parameters with respect to PFS (baseline values above and below median—HR = 2.96, p = 0.0009; baseline values above and below 75. percentile—HR = 2.44, p = 0.0062; normalization after the end of treatment—HR = 0.46, p = 0.0125; 50% reduction before IDS—HR = 0.25, p = 0.0017) and with respect to the OS (baseline values above and below median—HR = 1.88, p = 0.0087; normalization after the end of treatment—HR = 0.08, p = 0.0003; 50% reduction before IDS—HR = 0.39, p = 0.0469). In the multivariate analysis comparison of the well-established prognostic factors with the selected new factors, based on the HE4 measurements, the prolongation of PFS and OS was significantly influenced (HR = 0.29, p = 0.00008 and HR = 0.08, p = 0.00005, respectively) by the normalization of the HE4 marker after the end of treatment, that is, after the last course of chemotherapy, and a 50% reduction before IDS in the group treated with neoadjuvant chemotherapy significantly extended the time to disease progression (HR = 0.23, p = 0.0024).

## Discussion

Neoplastic marker HE4 is undoubtedly one of most popular markers studied by researchers with interest in biomarkers in gynecological oncology [7–10]. This protein was first identified in epithelial cells of the epididymis. This protein’s function, despite numerous studies conducted to date, has not been clearly established. However, it seems that among other things, the protein participates in the processes of cellular growth. Probably HE4 modulate protease activity, and it will affect a wide variety of cellular functions, since proteases are essential for many biological processes including growth factor signaling [11–12]. It is possible that HE4 from different tissues may preferentially exist in different forms, thus possessing different functions [13]. In neoplastic disease, it might additionally participate in the processes of adhesion, migration and the promotion of tumor growth [14]. Many studies corroborated the effectiveness of HE4 in the preoperative diagnostics of patients with ovarian tumors and verified its specificity, which is significantly higher than that of CA 125 [9,10,15].

Recent publications assessing the prognostic capabilities associated with this marker began to appear [16–20]. However, to date, such reports remain scarce, and there are only approximately 20 published studies, often on small groups of patients. The study group sizes varied from 8 to 275 women, while in only a small number of reports, the number of study participants was equal to or higher than 90 [16–35]. This fact clearly impacts the diversity of the results. However, all of the works underscore the statistically demonstrated value of HE4 as a prognostic factor [22–28]. In the analyses published to date, the authors utilize various laboratory techniques/tests (Fujirebio, Abbott, Roche) and diverse statistical methods, which may impedes a proper comparison of the results, influencing the absolute values of the marker itself, as well as the cut-off points set by the authors, which should not be adopted by other researchers who use different types of tests. Among the statistical methods, the most commonly applied tests include the Kaplan-Meier survival analysis, ROC curves and regression models with uni- or multivariate analysis. The majority of reports published to date are retrospective in nature. Taking all of those factors into consideration, it seems that there is still room for research on this problem. It appears that our prospective study, consisting of 90 patients and based on a relatively long follow-up time, might aid in determining the ultimate value of the HE4 marker as a prognostic factor. In our study, we conducted a large number of analyses using various cut-off points at different stages of the first-line treatment and utilized the majority of known statistical methods assessing the value of the studied parameter as a prognostic factor (Kaplan-Meier survival analysis, logistic regression models and receiver operating characteristics curve analysis). Using the ROC and AUC analyses, we demonstrated that the HE4 marker might be a good predictor of platinum sensitivity, complete clinical remission and surgical outcome. Other authors also utilized this statistical method [18,23,27,29,30]. Vallius et al. [29] only studied patients with advanced serous ovarian cancer subjected to neoadjuvant treatment (25 women). These researchers showed that an assessment of this marker before interval cytoreductive surgery (AUC = 0.877) was of greater diagnostic value than a preoperative evaluation. Our group of patients subjected to neoadjuvant chemotherapy were very similar to that studied by Vallius et al. [29], consisting of 42 patients with advanced serous ovarian cancer referred for neoadjuvant chemotherapy after a primary diagnostic laparoscopy or exploratory laparotomy. However, our results were different, and in this particular group, the predictive value of the HE4 marker was not statistically, significant, and the AUC value before cytoreductive surgery amounted to 0.452. However, in the PDS group, the prediction of surgical success was excellent (AUC = 0.846), as in the study by Angioli et al [30], demonstrating very similar results with a prediction of optimal cytoreduction using the HE4 marker, reaching an AUC = 0.861. Nassir et al. [23] published results demonstrating that the HE4 marker may predict disease recurrence within 12 months from the end of chemotherapy (AUC = 0.801), as well as during the first 6 months from the end of treatment (AUC = 0.719). In our study, it was also possible to assess platinum sensitivity using the HE4 protein, although the value of the area under curve was lower than in the cited publications (AUC = 0.644, p = 0.0351). In the studies by Braicu et al. [27] on the plasma HE4 concentration, the AUC for residual disease was 0.634. We also used another statistical method, a logistic regression model to test the predictive power of HE4 in this cohort. We found that HE4 protein can predict platinum sensitivity and DFS occurrence but not the surgical outcome and 2-years survival.

The assessment of survival using Kaplan-Meier curves and the log rank test is a popular method of evaluating the effectiveness of therapy and comparing the predictive factors. Using this statistical method, we showed that the time to progression was correlated with the preoperative HE4 values (shorter by at least several months in patients whose preoperative HE4 concentrations were above the median– 307 pmol/L, or the 75. percentile– 682 pmol/L, or without normalization of the marker after the end of chemotherapy). In our study, the overall survival also depended on the baseline HE4 values and the normalization after the last course of chemotherapy, but the analysis additionally revealed that the normalization of the marker values after the third course of chemotherapy, as well as a 50% reduction in the patients treated with neoadjuvant chemotherapy prolonged the overall survival by over 20 months. As in our study, many authors demonstrated a statistically significant correlation between the HE4 values and the PFS and OS [21,22,27,32,33]. In the majority of the cases, the authors analyzed the influence of HE4 on PFS and OS based on the preoperative concentrations of this marker [21,22,32,33]. Paek et al. [31] assumed the normal HE4 values were at the level of 70 pmol/L and observed that these values significantly influenced the PFS but not the overall survival. In their Kaplan-Meier survival analysis, Kaijser et al. [21] demonstrated the impact of the preoperative HE4 values on OS and PFS, although this influence was not confirmed in the multivariate analysis. Kong et al. [33] set the median of the preoperative HE4 concentrations and, based on this value, concluded that patients with protein levels above the median were characterized by a shorter time to disease progression. However, in their publication, the median value was very low and equaled only 98.7 pM, as opposed to our studies (307 pmol/L) and the studies by Trudel et al. [22] (394 pmol/L). In the study by Trudel et al. [22], the values above the median were associated with a significantly shorter time to progression (p = 0.0003) and overall survival time (p = 0.004).

The results of the Kaplan-Meier survival analysis and log rank test were additionally compared in the univariate and, above all, the multivariate logistic regression analysis. In the univariate analysis, we corroborated the influence of the preoperative values and the normalization of the HE4 marker after the end of chemotherapy on PFS. Moreover, in this analysis, the 50% reduction before the interval cytoreductive surgery was significantly correlated with the time to disease progression. However, in the multivariate analysis, compared with the standard prognostic values, the following factors influenced the time to progression, including the normalization of the HE4 marker after the last course of chemotherapy and the 50% reduction before interval surgery (IDS). Similar results were obtained for overall survival, but the multivariate analysis statistical significance was confirmed only for the normalization of the marker levels after the end of the first-line treatment (HR = 0.08, 95%CI: 0.02–0.27, p = 0.00005). As in our studies, other authors also demonstrated the prognostic value of the HE4 marker in both the uni- and multivariate analysis. Regarding the Kaplan-Meier analysis, the majority only studied the influence of the preoperative HE4 levels [21,22,25,29,31–33]. However, Steffensen et al. [25] examined the influence of the HE4 values measured immediately after surgical treatment but before the commencement of chemotherapy on the survival parameters of the patients with ovarian cancer. These researchers demonstrated that the HE4 value before first-line chemotherapy was an independent prognostic factor, and values above the median correlated with a shorter time to progression (HR = 1.77, p = 0.04) and overall survival time (HR = 3.17, p = 0.005). Vallius et al. [29] studied a group of women treated with neoadjuvant chemotherapy and demonstrated that surgical outcome correlated with the concentrations of the marker measured immediately before IDS, not at the time of diagnosis. Moreover, a reduction over 80% exerted a statistically significant influence on the overall patient survival. The authors suggested that HE4 concentrations before IDS might be used to decide on the implementation of cytoreductive surgery or second-line chemotherapy. Recent studies by Nassir et al. [23] showed that the assessment of the HE4 marker during follow-up after treatment might successfully predict disease relapse within the first 12 months from treatment.

In the majority of reports, there is a consensus as to the prognostic significance of serum HE4 measurements in the preoperative period [21,22,31,33–35], although a small number of studies performed to date corroborate the significance of HE4 levels assessed in the postoperative period [25], after first-line chemotherapy [29] or during follow-up after treatment [23]. This topic certainly requires broader investigation based on an analysis of a large study group. However, given the short time period in which the marker has been officially approved for the diagnostics and monitoring of treatment, large prospective studies and meta-analyses are expected in the coming years.

The main novelty lies not only in the assessment of pre-operative HE4 levels, but also in the assessment of HE4 concentrations at different times during the first line therapy and its relationship with the response to treatment. It seems that a very important result of our work is the fact that PFS and OS are affected not by the initial HE4 value but by the fact that it normalized after the first line chemotherapy, which has not been described so far. The significance of 50% reductions in the HE4 concentration before the interval debulking surgery has not been described yet.

## Conclusions

Based on the conducted studies and literature review, it seems that regular measurements of the HE4 marker during the first-line treatment of ovarian cancer may be of prognostic significance for patients. Preoperative HE4 values are also associated with the outcome of surgical treatment and platinum sensitivity. The normalization of HE4 marker levels after the end of treatment and a reduction of HE4 concentrations by 50% before interval cytoreductive surgery are strong predictive factors with regard to time to progression, as well as overall survival time, corroborating the value of HE4 as an independent prognostic factor.

## Supporting information

S1 Table Database. (PDF)
