# Supplementary material for: Could HE4 level measurements during first-line chemotherapy predict response to treatment among ovarian cancer patients?
Source: PLoS One. 2018 Mar 27;13(3):e0194270. doi: 10.1371/journal.pone.0194270 (PMC5870956; doi:10.1371/journal.pone.0194270)
Supplement: S1 Table — (PDF) [file pone.0194270.s001.pdf]

		Age	M/PM	ADJ/NEO/	FIGO
		72	M	ADJ	IIIC
		66	M	ADJ	IIIC
		68	M	ADJ	I
		54	M	ADJ	IIA
		31	PM	ADJ	IA
		54	M	ADJ	IIIC
		71	M	ADJ	I
		68	M	ADJ	IIA
		60	M	ADJ	I
		65	M	ADJ	IC
		58	M	ADJ	II
		39	PM	ADJ	III
		65	M	ADJ	IC
		74	M	ADJ	IIIC
		64	M	ADJ	IIA
		58	M	ADJ	IIA
		54	M	ADJ	IIIC
		52	M	ADJ	IIIB
		50	M	ADJ	IIIC
		64	M	ADJ	I
		69	M	ADJ	IIIC
		81	M	ADJ	III
		55	M	ADJ	II
		58	M	ADJ	III
		52	M	ADJ	III

		56	M	ADJ	IA
		52	M	ADJ	I
		66	M	ADJ	III
		53	M	ADJ	IC
		59	M	ADJ	IIA
		52	M	ADJ	I
		62	M	ADJ	I
		40	PM	ADJ	IA
		52	M	ADJ	IIIC
		53	M	ADJ	IIIC
		72	M	ADJ	
		61	M	ADJ	III
		66	M	ADJ	I
		63	M	ADJ	I
		62	M	ADJ	I
		46	M	ADJ	II
		61	M	ADJ	IC
		50	PM	ADJ	I
		57	M	NEO	IIIC
		69	M	NEO	IIIC
		82	M	NEO	IV
		58	M	NEO	IIIC
		63	M	NEO	IIIC
		65	M	NEO	IIIC
		80	M	NEO	IIIC
		71	M	NEO	IIIC
		85	M	NEO	IV

		57	M	NEO	IIIC
		63	M	NEO	IV
		50	PM	NEO	IIIC
		41	PM	NEO	IIIC
		51	PM	NEO	IIIC
		79	M	NEO	III
		59	M	NEO	IIIC
		55	M	NEO	IIIC
		49	PM	NEO	IIIC
		73	M	NEO	IIIC
		56	M	NEO	IIIC
		63	M	NEO	IIIC
		57	M	NEO	IIIC
		84	M	NEO	III
		60	M	NEO	IIIC
		52	M	NEO	IIIC
		57	M	NEO	III
		64	M	NEO	III
		70	M	NEO	III
		38	PM	NEO	III
		57	M	NEO	III
		52	M	NEO	III
		87	M	NEO	III
		78	M	NEO	III
		58	M	NEO	III
		82	M	NEO	III
		80	M	NEO	III

		52	M	NEO	III
		52	M	NEO	III
		63	M	NEO	III
		55	M	NEO	III
		53	M	NEO	III C
		47	PM	NEO	III
		62	M	ADJ	II
		53	M	ADJ	IC
		56	M	ADJ	I
		68	M	ADJ	III
		44	PM	ADJ	II

HIST-PAT	GRADING	RESIDUA	RESIDUAL_IDS[	Optimal/Suboptimal	HIST_PAT after IDS
serosum	2	15	-	S	-
serosum	2	0	-	O	-
serosum	2	20	-	S	-
serosum	3	0	-	O	-
mucinosum	1	0	-	O	-
serosum	?	20	-	S	-
serosum	?	0	-	O	-
serosum	3	15	-	S	-
mucinosum	3	0	-	O	-
mucinosum	1	0	-	O	-
endometric	2	0	-	O	-
serosum	1	0	-	O	-
serosum	3	0	-	O	-
serosum	3	2	-	S	-
serosum	2	0	-	O	-
serosum	3	0	-	O	-
serosum	2	20	-	S	-
serosum	3	10	-	O	-
serosum	3	20	-	S	-
serosum	1	0	-	O	-
serosum	3	15	-	S	-
serosum	3	20	-	O	-
endometric	3	0	-	O	-
serosum	3	10	-	O	-
serosum +	3	20	-	S	-

serosum	3	0	-	O	-
serosum +	3	0	-	O	-
serosum	3	0	-	O	-
clear cell	2	0	-	O	-
serosum	3	0	-	O	-
serosum	3	0	-	O	-
Brener typ	1	0	-	O	-
serosum	1	0	-	O	-
serosum	1	0	-	O	-
serosum	2	50	-	S	-
serosum z	2	0	-	O	-
serosum	3	15	-	S	-
mucinosun	1	0	-	O	-
serosum	1	0	-	O	-
mucinosun	1	10	-	O	-
endometri	2	0	-	O	-
serosum	2	0	-	O	-
serosum	2	0	-	O	-
serosum	3	-	0	O	tak
serosum	1	-	0	O	tak
serosum	2	-		S	tak
serosum	3	-	0	O	tak
serosum	3	-	1	O	tak
serosum	2	-	20	S	tak
serosum	3	-	2	O	tak
serosum	2	-	0	O	tak
serosum	3	-			-

serosum	2	-	20	S	tak
serosum	3	-	30	S	tak
serosum	-	-	20	S	tak
serosum	2	-		O	tak
serosum	2	-	10	O	tak
serosum	3	-	20	S	nie
serosum	2	-	20	S	tak
serosum	1	-	30	S	tak
serosum	1	-	10	O	tak
mucinosun	1	-	10	O	tak
serosum	3	-	10	O	tak
serosum	2	-	10	O	tak
serosum	3	-	10	O	tak
serosum	3	-	0	O	tak
serosum	3	-	10	O	tak
serosum	3	-	10	O	tak
serosum	3	-	10	O	tak
serosum	3	-	10	O	tak
serosum	3	-	10	O	tak
serosum	-	-	10	O	nie
serosum	3	-	10	O	tak
serosum	3	-	10	O	tak
serosum	3	-	0	O	nie
serosum	2	-	20	S	tak
serosum	3	-	20	S	tak
serosum	3	-	20	S	tak
serosum		-	10	O	tak

serosum	3	-	20	S	tak
		-	20	S	TAK
serosum	2	-	15	S	tak
endometric	3	-	10	O	TAK
serosum	3	-	10	O	TAK
serosum	3	-	0	O	-
serosum	1	0	-	O	-
mucinosum	1	0	-	O	-
serosum	3	0	-	O	-
serosum	3	5	-	O	
serosum	2	0	-	O	-

HE4 >/< 500	CA 125 >/<500	Before PDS and lap		Before IDS	
W/N	W/N	HE4	CA125	HE4	CA125
N	N	380,57	262,3		
N	N	245,9	321,9		
N	N	65,9	20,4		
N	N	123,5	87		
N	N	68,1	114,2		
N	W	208	535,1		
N	N	156,4	50,36		
N	W	345	543		
N	N	109,8	47,8		
N	N	246,1	57,9		
N	N	65,75	41,5		
N	N	35,79	33		
N	N	342,9	111,24		
W	N	935,4	432		
N	N	83,7	34,4		
N	N	221	73,9		
W	W	1500	1318		
N	N	211,6	325		
W	W	1500	1393		
N	N	152,7	135,5		
W	W	1298	537		
N	N	198	15,9		
N	N	121	60,97		
W	N	850	339		
N	N		86,15		

N	N	77,9	339,4		
N	N	50,03	45,59		
W	W	499,2	639,8		
N	N	179,9	32,7		
	N		170		
N	N	41,1	70,5		
	N	188	1441		
N	N	49,9	75		
N	N	283,5	310		
	W	311	633,2		
N	N	239	98		
W	W	543,2	905,1		
N	N	48,4	11,3		
N	N	43	16,5		
N	N	138	110,2		
N	N	120,7	399		
N	N	320	115,4		
N	N	233	58		
W	W	1448	525,5	67	25
W	W	658,4	745,1	59,8	28,5
W	W	789	580	324	121
W	W	1372	1290	278,3	41
W	W	1500	14199	834,6	9642,2
N	N	389,9	361,03	166	88,7
W	W	871,2	727,5	404	191
N	W	324,5	1636	268,1	42,3
N	N	134,6	287		

N	N	53,9	201	144	245
N	W	2556	2810	458,7	1207
N	W	318,6	683	152,4	114,4
N	N	465	86,9	89	19,7
W	W	1116,27	1639,6	60	33
W	N	223	176	56	13
W	W	1057	500	231	237
W	W	543	735	208	358
N	N	111	36,5	43	12
N	N	226	184	67	19,1
W	N	543,5	189.6	102	337,9
N	N	102,4	199	70,8	5,9
W	W	521	537	261,4	60
W	N	612	381	274,4	24,2
N	N	53,1	102	39	5,6
W	W	639	523	68,2	11,5
W	W	1197	1584		33,8
N	W	195	2534,2	85,2	218,7
W	W	960,7	536	93,9	20,5
W	N		487,1	32,3	7,1
W	N	1500	301,6	141,3	10,5
W	W		521,8		23,7
N	W	374	778	218	122,2
W	W	701,2	600	205	119
W	W	1500	3832	341	726
W	W	1500	10000	341	211
W	W	3608	5659,3	433	773

W	W		1061		61
N	N	273	191	273	238
N	N	307,1	448,1	55,6	28,3
W	W	483	2996	246,7	736,2
W	W	682,2	769	84,7	63,2
W	W	876,8	4638,8	321	123
N	N	113,6	72,1		
N	N	89,9	227,6		
N	N	137,8	215		
W	N	516,5	190,5		
N	N	104,5	198,1		

50% decrease before IDS		50% decrease aftrer PDS		I course		II course
HE4	CA 125	HE4	CA125	HE4	CA125	HE4
		T	T	77,1	10,1	64,1
			T	69	9,4	61,4
		T	T	42,7	40,3	46,1
			T	67	45	68
		N	N	203	529	154
		T	T	56,5	22,3	68,4
		T	T	87,8	223,1	84,6
		N	T	60,5		58,2
		T	N	71,7	43	69,1
				65,8		73,6
				67	25	65
		T	T	76,1	47	63,6
		T	T	101,6	22,8	83,4
				83,7	25,2	92,1
		T	T	98	36	77
		T	N	647	769,2	435,9
		T	T	34,3	20	33,6
		T	T	330	56,4	243,7
		N	N	83		90,6
		N	T	657,6	145,1	463,4
		N		116,7	11,4	109,8
		T	N	60,3	33	60,3
		T	N	343	224	298
			N	48,7	68,3	65,1

		T	T	56,7	10,7	59,2
			N	48,3	8,5	48,6
		T	T	54,4	24,9	72,8
		T		71,5	29,8	77,4
			T	43,5	12,3	52,2
			T	40,3	10	57,5
		T	T	60,9	15,2	74,4
			T	43,7	10,5	53,6
		T	T	65,9	53,1	62,3
		N	N	186,3	812	56,3
		T	T	79	26,1	85,7
		T	T	264,1	289	75,1
				42,5	11,3	48,2
				38,4	15,5	42,1
		N	N	100,7	224,5	114,1
		T	T	40,3	26,5	44,9
		T	T	52,4	14	66,9
		T	T	48,8	27,9	56,9
T	T					
T	T			755,4	695,2	276,4
T	T					
T	T			1500	1290,7	-
N	T			1500		715,3
T	T					
T	T					935,9
N	T				1636,8	172,6
				111,9	54,4	108,2

N	N					449,8
T	T			634,8		937,3
T	T			423,8	815,4	266,7
T	T			181,3		110,1
T	T					
T	T					
T	T					
T	T					
T	T					
T	T					
T	T			671,7		194,2
N	T			96,6		80,3
T	T			112,7		73,2
T	T			555,6	109,6	
-	T			53,1	19,6	42,3
T	T			640,5	255,2	140,5
T	T			144	183,3	112,9
T	T			88	238,8	
T	T			1003	629	184
T	T			64,8	219,9	33,7
T	T			6998	389,9	1502,4
	T			718	313	555
N	T			48,7	68,3	234,1
T	T			382	593	176
T	T			328	421	294
T	T			1421	21000	569
T	T			2924	1714	1927

	T					
N	N			187,1	191	236
T	T			614	527	159
N	T			660,5	2581	194,9
T	T			758,3	1917,9	261,7
T	T			-	-	-
		T	T	-	-	87,8
		N	N	91,9	11,9	81,1
		N	N	108,5	217	47,8
		T	T	67,9	52,1	73
		T	T	44,7	52,2	86,2

	III course		NORMALISATION aftre 3rd course		IV course		V course
CA125	HE4	CA125	3 HE4	CA125	HE4	CA125	HE4
	63,6	9,7	T	T	62,3		70,9
	69,1	11	T	T	70,6	11,2	83,2
	46,8	34,9	T	T	46,9		45,3
	72	11	N	T	55		65
	108,8	17,7	N	T	127,8		158,1
	66,2	17,7	T	T	70,4		76,4
	72,6	71,7	N	N	73,3		73,1
	56,2	33,6	T	T	56,2	33,6	52,8
	74,3	14,9	N	T	88,9		89,3
	77,7	5,2	N	T	78,8		82,7
	67	9,5	T	T	46		46
	58,3	28	T	T	59,3		36,3
	61,5	38	T	N	58,6	8,5	64,4
	89,9	12,7	N	T	114,1		103,6
	53,5	18	T	T			46,1
	336,1	199,4	N	N			257
	32,4	7,2	T	T	33,5		33,3
	215,8	198,5	N	N	180,4		143,5
	86,8	7,7	N	T	99,6		125,7
190,9	220,2	132,4	N	N	106,2		96,9
	109,1	10,7	N	T	128	11,1	123,9
	63,2	24	T	T	63		58,2
	174,9	308,4	N	N	92,3		79,5
	63,3	10,5	T	T	61,2	7,8	54,8

24	58,7	9,5	T	T	59,8		60,1
	47,3	7,8	T	T	52,6		48,1
9,6	83,4	11,1	N	T	70,1	8,5	62,5
16,8	60,6	14,7	T	T	66,9	14	62,1
8,2	60,4	7,3	T	T	67,2		59,4
9	59,8	10,9	T	T	61,9	10,5	57,5
	70,1	25,3	N	T	64,5		64,8
7,5	47,7	6,4	T	T	47,4	7,1	38,4
35,3	69	28,1	T	T	54,7	22,9	62,8
	59,8	47,1	T	T	52,5	13	56,9
	76,5	14,9	N	T	69,7	12,2	68,1
	47,1	30	T	T	42,6	11,5	40,5
10,6	56,1	11,5	T	T	52,5	5,9	48
20,7	37,1	10,5	T	T	50,2	10,3	47,5
43,5	198	45	N	T			
	43	22	T	T			
14,7	55,9	12,4	T	T	53,7	10,5	59,2
	60,4	10,3	T	T	66,1	11,3	60,4
	69	28			67		59
	134,9	190,9			81		55,1
		121,8				48	
	863,2	457,4			336,3	N	129,6
	168,9	1097,1			66		67
	181,7	29			166	88,7	150,1
	582,6	423,1			725,3	471,9	404,8
	155	153,9			161,9	139	ODMÓWI
	112,2	43					

1175,9	208,6				144,9	245,7	140,6
	876					1207	
	200,3	154,5			68	21,2	64
	90,9	128,8			89	19,7	35,5
		9,2					50,8
		>10000				>10000	
	322,3	483			208,9	358,2	206,8
	81,5	6,3					76,5
	124,8	624			107,5	624	58,7
	72	6					70,8
	57,3	54,4			55	33,4	54,2
	367,8	25,6			196,5	16,4	202
	39	5,6			46,2		37,4
	91,2	18,2			68,2	11,5	56,1
183,3	92,3	57,3			96,6	61,7	96,4
	85,2	218,7			83,2	426,3	
176,7	93,9	20,5			268,6		
	32,3	7,1			29,2		
188,5	340,2	16,6			242,2	11,3	175,9
	386	104			276		238
	203,6	292,6			210,2	212,7	
					130	453	113
484	257	395			253	385	
	343	1739					341
1080	703	621			435	907	390

						89,4	
	225	230,5			257,1		299,8
368	64	61			45,6		5,1
1066,2	117,9	345			97,9		69,8
-	136,3	269,8			110,7	-	90,8
-	-	-			-	-	-
19	112,1	18,7			94,6	17,5	106,1
11,9	73,2	9,3			78,7	7,7	84,3
32,7	51,9	9,6			53,4	11,2	52,5
31,6	73	31,6			62,4	10,9	66,3
16,3	76,1	9,7			73,8	8,4	67

	VI course		VII course		VIII course		IX course		X course
CA125	HE4	CA125	HE4	CA125	HE4	CA125	HE4	CA125	HE4
	144,9	258,6							
	60,3	9,8							
	78,6	10,6							
	45,3	32,8							
	67,5	33							
	172,4	15,7							
	73	18,6							
	69,5	45,1							
27,9	48,4	35,7							
16,2	90	22,3							
	76,3	9,7							
		11,6							
27,6	68,6	27,6							
	66,6	8,7							
	96,5	14,4							
	56,1	13,8							
	250,8	87,6							
8,5	39,4	8							
	509,3	81,5							
	125,4	7,1							
	78	13,4							
	122	11,1							
	59,3	14,6							
	70,8	16,9							
10,4	53,4	11,4							

	61	9,3							
	49,9	8,2							
10	61,9	8,2							
	395,8	410,2							
	66,6	13,7							
10,4	45,1	10,8							
	63,7	16,8							
8,1	38,6	8,3							
22,2	69,4	22,4							
9,6	56,5	12,3							
	68,8	15,8							
	43,5	6,3							
8,6	52,5	5,9							
17,7	46,8	17,1							
11,2	59,6	9,2							
13,6	57,3	11,1							
	64,1	17	64,4		42		52,3	6,7	
28,5	49,3	23,6	52,8		50		56,1	19,2	60,3
51,1							64,8	11,4	54,1
43,6	123,4		107,3		108,2		107,8	28,8	
48,4	71,5	37,2	73,4		75,4		90,8	30,7	
	175	78,3	238,1		220				
191,1	309		196,1	58,8	287		269	76,8	267,4
ODMÓWIŁA DALSZEGO LECZENIA									
-	-	-	-	-	-	-	-	-	-

	150,7	316,2	140	379,5	204,3	362,2	216,2	421,5	325
		627			458,7	624,1	634,8	507,6	937,3
	64,2	9	58,2		56,6		56,7	3,2	
14,6	34,6	13,3	41,2		38,9		44,2	16,2	
		33	59,3	19,7	51		52,7		55,8
	41,2								
1390,5	231	237,9		210	71,8	157,1	60,4	107,7	
	193,4	161,5	144,7		120,7		129,7	82,7	86,6
				22,1	64,5		61,2		
	81,5	6,3	82,1		82,1		75,7		81
26,4	52		54,6		48,1		49,1		46,8
	67,7		70,2	6	73,7		76	4,7	76
	60,8	15,7	52,3		54,8		66,2		58,2
16,7			169	15	246	39,2	307	23,9	
3,1	40,1	5,2	49	4	37,9	4,3	40,2	4,8	
	91,2		50,7	7,4	53,5		53,9		50,7
47,5	88,9	72,4							
480,1	186,4	480,1	372,1	859,3	676,4	1869,3	673	ascites i odstapionc	
3,5	97,9	5,4	30		31,8		33,4	5,6	
10,9	98,8	7	110,7	10,6	93	6,8	98,7	5,6	88,1
19,5	210	15,6							
	169,6	144,4			183,3	111,4			
406	102	293	109		105	276	95	332	
	341	726							
211	146	207	175		147	146			
722	433	773							

61,9									
41,9									
7,3	46,7	5,9	47,5	7	44,7	7,7			
28,7	76,4	23	61,4	19,2	64,2	22,1			
79,8	89,5	78,8	98,7	80,7	93,5	70	84,1	59,4	-
-	-	-	-	-	-	-	-	-	-
-	105,5	27,2							
8,9	81,5	8,2							
8,2	54,5	9,6							
10,5	69	10,5							
9,3	73,8	8,4							

	After chemothrapy		Normalisation aftrer cht	
CA125	HE4	CA125	HE4	CA125
	144,9	258,6	N	N
	55,4	10,43	T	T
	83,2	10,2	N	T
	45	32,8	T	T
	65	9,9	T	T
	241	22,7	N	T
	78,1	16	N	T
	68	33,7	T	T
	58,4	30,5	T	T
	90	22,3	N	T
	76,3	6,8	N	T
	47,4	8,6	T	T
	68,6	27,6	T	T
	66,6	10	T	T
	85,4	13,9	N	T
	44,4	14,4	T	T
	234,3	78,4	N	N
	33,3	8,5	T	T
	330	55,4	N	N
	99,6	7,7	N	T
	64	10	T	T
	137	10,9	N	T
	59,3	14,6	T	T
	106	14,5	N	T
	52,8	10,5	T	T

	58	9,6	T	T
	47	6,2	T	T
	55,9	6,5	T	T
	414,3	341,5	N	N
	66,6	13,7	T	N
	39,4	7,5	T	T
	67,5	15,6	T	T
	37,3	8,9	T	T
	62,8	22,2	T	T
	49,3	9,8	T	T
	83,4	6,7	N	T
	123,9	189	N	N
	52,5	5,9	T	T
	46,8	17,1	T	T
	106,3	13,8	N	T
	60	9,8	T	T
	57,2	11,1	T	T
	56,4	7,1	T	T
18,4	86,2	177,4	N	N
	72	24,5	N	T
	157,8	123,7	N	N
	90,8	30,7	N	T
	345	123	N	N
	267,4	83,2	N	N
	393,9	41,6	N	N
-	-	-		

546,3	1110	1325,6	N	N
	1500	2416,5	N	N
	72,8	13,5	N	T
	50,2	18,3	T	T
24	51,5	12,3	T	T
	57,8	8	T	T
	60,4	107,7	T	N
	126,8	38,3	N	N
	69,7	164	T	N
30,7	81	39,7	N	N
11,8	45	10,4	T	T
4,2	73	5,6	N	T
35,8	80,6	50,6	N	N
	307	23,2	N	T
	39	6,8	T	T
7,4	53,5	7,6	T	T
	59,2	44,4	T	T
odstapiono od chemioterapii				
	123	77	N	N
	31,8	3,5	T	T
4,7	78	5,6	N	T
	210	15,6	N	T
	218,1	122,2	N	N
	69	232	N	N
	221	321	N	N
	167	98	N	N
	543	456	N	N

	321	76	N	N
	341	77	N	N
	48,2	7,5	T	T
	66	28	T	T
-	87	65	N	N
-	-	-		
-	105,5	27,2	N	T
-	76	7	N	T
-	51	9,8	T	T
-	5,9	9,2	T	T
-	68	10	T	T

PFS Y/N	PFS	Recurrence	tinum sensiti	Death	OS	2YS Y/N
			Niewrażliwe			
N	2		N	TAK	9	N
T	47	N	W	NIE	47	T
N	4		N	TAK	9	N
T	48	N	W	NIE	48	T
N	4		N		13	N
T	10	T	N	TAK	36	T
T	46	N	W	NIE	56	T
T	13	T	W	NIE	53	T
T	48	N	W	NIE	48	T
N	5	N	N	TAK	9	N
T	46	N	W	NIE	46	T
T	50	N	W	NIE	50	T
T	17	T	W			T
T	42	T	W	NIE	42	T
T	46	N	W	NIE	46	T
T	47	N	W	NIE	46	T
N	2		N	TAK 30.12.1	37	T
T	43	N	W	NIE	43	T
N	5		N	TAK 10.13	25	T
T	12	T	W	TAK 2014	33	T
T	18	T	W	NIE	42	T
T	21	T	W	TAK 05.15	40	T
T	40	T	W	NIE	40	T
T	9	T	N	TAK 2012	16	N
T	34	N	W	NIE	34	T

T	31	T	W	NIE	31	T
T	38	N	W	NIE	38	T
T	30	N	W	NIE	30	T
T	9	T	N	TAK		N
T	26	T	W	NIE	31	T
T	30	N	W	NIE	30	T
T	41	N	W	NIE	41	T
T	32	N	W	NIE	32	T
T	29	N	W	NIE	29	T
T	27	T	W	NIE	35	T
T	34	N	W	NIE	34	T
T	14	T	W	NIE	36	T
T	29	N	W	NIE	29	T
T	28	N	W	NIE	28	T
N			N	TAK	6	N
T	31	N	W	NIE	31	T
T	31	N	W	NIE	31	T
T	31	T	W	N	36	T
T	18	T	W	TAK	30	T
T	18	T	W	TAK	24	T
T	40	N	W	NIE	40	T
T	12	T	W	TAK	44	T
T	14	T	W	TAK	22	N
N	8		N	TAK	16	N
N	3		N	TAK	11	N
N			N	TAK	16	N
N	3		N	TAK	3	N

N	4	T	N	TAK	13	N
N	6		N	TAK	15	N
T	26	T	W	NIE	45	T
T	36	T	W	TAK	47	T
T	35	T	W	NIE	53	T
T	13	T	W	NIE	53	T
T	15	T	W	NIE	54	T
T	17	T	W	NIE	52	T
T	35	T	W	NIE	54	T
T	14	T	W	TAK	27	T
T	12	T	W	NIE	43	T
T	22	T	W	TAK	45	T
N	7		N	TAK	18	N
N	N	T	N	NIE	18	N
T	17	T	W	NIE	39	T
T	39	N	N	NIE	39	T
T	19	T	W	NIE	54	T
N	6		N	TAK	12	N
N	3	T	N	TAK	6	N
T	26	T	W	NIE	45	T
T	21	T	W	NIE	37	T
T	17	T	W	TAK	48	T
T	12	T	W	TAK	22	N
T	13	T	W	TAK	37	T
N	4		N	TAK	13	N
N	9		N	TAK	30	T
N			N	TAK	13	N

N	5		N	TAK	18	N
N	5		N	TAK	10	N
T	36	T	W	NIE	38	T
T	13	T	W	TAK	21	N
T	10	T	N	TAK	21	N
T	37	N	W	NIE	37	T
T	34	N	W	NIE	34	T
T	36	N	W	NIE	36	T
T	32	N	W	NIE	32	T
T	23	N	W	NIE	23	T
T	30	N	W	NIE	30	T
